# Nanopattern surface improves cultured human myotube maturation

**DOI:** 10.1186/s13395-021-00268-3

**Published:** 2021-05-05

**Authors:** Jessica Brunetti, Stéphane Koenig, Arthur Monnier, Maud Frieden

**Affiliations:** Department of Cell Physiology and Metabolism, University of Geneva, Geneva, Switzerland

**Keywords:** Human primary myoblasts, Cell alignment, Myotube maturation, Acetylcholine receptor clusters, Ca^2+^ signals

## Abstract

**Background:**

*In vitro* maturation of human primary myoblasts using 2D culture remains a challenging process and leads to immature fibers with poor internal organization and function. This would however represent a valuable system to study muscle physiology or pathophysiology from patient myoblasts, at a single-cell level.

**Methods:**

Human primary myoblasts were cultured on 800-nm wide striated surface between two layers of Matrigel, and in a media supplemented with an inhibitor of TGFβ receptor. Gene expression, immunofluorescence, and Ca^2+^ measurements upon electrical stimulations were performed at various time points during maturation to assess the organization and function of the myotubes.

**Results:**

We show that after 10 days in culture, myotubes display numerous functional acetylcholine receptor clusters and express the adult isoforms of myosin heavy chain and dihydropyridine receptor. In addition, the myotubes are internally well organized with striations of α-actinin and STIM1, and occasionally ryanodine receptor 1. We also demonstrate that the myotubes present robust Ca^2+^ responses to repetitive electrical stimulations.

**Conclusion:**

The present method describes a fast and efficient system to obtain well matured and functional myotubes in 2D culture allowing thorough analysis of single-cell Ca^2+^ signals.

**Supplementary Information:**

The online version contains supplementary material available at 10.1186/s13395-021-00268-3.

## Background

Skeletal myofibers are large multinucleated cells with an exceptional level of internal organization dedicated to produce strength upon nerve activation. Acetylcholine, released from nerve terminals, induced membrane depolarization (action potential) that is sensed by voltage-gated Ca^2+^ channels, Cav1.1, also called dihydropyridine receptor (DHPR). The conformational change of DHPR is transmitted to the ryanodine receptor 1 (RyR1), a Ca^2+^ channel localized at the terminal cisternae of sarcoplasmic reticulum (SR). The opening of RyR1 leads to a strong release of Ca^2+^ from the SR and eventually muscle contraction, an overall process called excitation–contraction (EC) coupling. Because of the size and organization of muscle fibers, and even if important progresses were accomplished over the years, it remains challenging to obtain well differentiated and matured myofibers *in vitro*. Several models of myogenesis were designed to generate muscle tissue that could be eventually engrafted in damaged/diseased muscles [1], to provide suitable models to study pathophysiological mechanisms of the disease [2, 3], or to understand the various steps of muscle formation [4, 5]. The sources of cells used to perform *in vitro* culture are diverse, such as C2C12 mouse cell line or primary myoblasts of murine or human origin. As well, cultures derived from human iPSC are used with promising outcomes [6, 7]. In the recent years, several studies have highlighted the benefits of establishing 3D compared with 2D culture. Indeed, 3D approaches promote the level of maturation and contractility of myotubes/myofibers and allow to keep cells longer in culture. The improved internal architecture, the presence of acetylcholine receptor (AChR) clusters, and the higher contractility are among the main advantages of 3D versus 2D culture [8]. However, 2D cultures are likely more appropriate for single-cell level analysis, but myotubes differentiated on 2D frequently do not mature properly with often a lack of striations and the appearance of small/broken cells [6] [8]. One cue demonstrated to promote a higher level of myotube maturation is the cell alignment. Indeed, skeletal muscles are composed of very long and aligned fibers, an organization that could be recapitulated, at least partially, in culture. To promote such alignment, self-fabricated substrates are usually created, which are nonetheless complicated to establish and require special lab equipment [9–11].

In the present study, we describe an *in vitro* maturation system that uses striated surface to culture human primary myoblasts. We report evidence of clear beneficial effects of growing and differentiating muscle cells on a striated surface, both at structural and functional levels. We show that on this surface, myocytes display rapidly a high level of internal organization and numerous AChR clustering. Finally, this culture model allows thorough analysis of single-cell robust and repetitive Ca^2+^ transients elicited by electrical field stimulations.

## Methods

### Cell culture

Human primary myoblasts were isolated from semitendinous muscle samples obtained after orthopedic surgery (surgical waste) on patients without known muscular diseases. All samples were collected anonymously after obtaining a written consent and approval by the University of Geneva (protocol CCER no. PB_2016-01793 (12-259) accepted by the Swiss Regulatory Health Authorities and approved by the “Commission Cantonale d'Ethique de la Recherche” from the Geneva Cantonal Authorities, Switzerland). The purification of myoblasts was performed as previously described [12].

Myoblasts were seeded either on a conventional dish with a flat surface (FluoroDish^TM^, Cat n°FD34-100; WPI), or on a nanopattern surface (Anisotropic Nano-Fabricated Surface, ANFS, Nanosurface Biomedical), that has ridges and grooves of 800-nm and 600-nm deep. Both surfaces were coated with Matrigel (Corning Matrigel Basement Membrane Matrix Growth Factor Reduced, Phenol Red Free, #356321) diluted at 1:100 in Ham's F-10 (ThermoFisher) as previously described [13]. Cells were expanded in a growth medium (GM; see Table 1), and when they reached around 90% of confluency, the differentiation was triggered by replacing GM with a differentiation medium (DM; see Table 1). At the time, cells were placed in DM, and during the first 3–4 days in differentiation, the medium was supplemented with 10 μM of TGFβ receptor I inhibitor (SB431542, #S4317, Sigma). After 4 days in differentiation, the TGFβ inhibitor was removed, and a thick layer of Matrigel diluted at 1:3 in DM was added to limit cell detachment [13], and then half of the medium was changed every 2 days.

**Table 1:** Cell culture media

	Concentration	Reference
**Growth medium (GM)**		
F-10 (Ham’s)		ThermoFisher 31550-023
BSA	0.5 mg/ml	Sigma 05482
Insulin	0.04 mg/ml	I-9278
Creatine powder	1 mM	Fluka 27900
Dexamethasone	0.39 μg/ml	Sigma D1756
Gentamycin	5 μg/ml	Sigma G1272
EGF	10 ng/ml	Corning 354001
FBS	15%	Lot 4205662K
Fetuin	0.5 mg/ml	Sigma F-3385
Sodium pyruvate	100 μg/ml	Sigma P4562
Uridine	50 μg/ml	Sigma U3003
**Differentiation medium (DM)**		
DMEM		Thermo Fisher 41965-039
BSA	0.5 mg/ml	Sigma 05482
Insulin	0.01 mg/ml	Sigma I5500
Creatine powder	1 mM	Fluka 27900
Gentamycin	10 μg/ml	Thermo Fisher 15710-049
Sodium pyruvate	100 μg/ml	Sigma P4562
Uridine	50 μg/ml	Sigma U3003
Horse serum	1%	Amimed 2-05F26-I

### Immunofluorescence

Human myotubes were fixed in PBS with 4% paraformaldehyde, permeabilized and blocked in PBS containing 0.3% of Triton X-100 and 5% goat serum. Proteins of interest were revealed by incubation of specific primary antibodies overnight at 4 °C, followed by incubation of fluorophore-conjugated secondary antibodies for 75 min at room temperature. Primary and secondary antibodies and their dilution are listed in Table 2. Nuclei were stained using ProLong® Gold Antifade Reagent with DAPI (ref. P36931, Life Technologies). To detect acetylcholine receptors, α-bungarotoxin (α-BTX, B13422, Invitrogen) was added together with the secondary antibodies. Images were acquired either with a widefield AxioImager M2 microscope (widefield microscopy, Zeiss, Germany) through a 20× objective (EC Plan-Apochromat 20×/ 0.8), or with a confocal Nikon A1r spectral microscope (Nikon; Japan) through a 60× objective (1.4 CFI Plan Apo Lambda). Three random fields were acquired for each dish, with a minimum of two dishes per experimental condition. Clusters of AChR bigger than 5 μm^2^ were considered for analysis. Analysis of the images were performed using ImageJ software. For display purposes, some pictures were rotated and rescaled to have the same resolution.

**Table 2:** List of primary and secondary antibodies

	Dilution	Reference
**Primary antibodies**		
Mouse anti-(sarcomeric) α-actinin	1:500	Sigma A7811
Mouse anti-Pax7	1:100	DSHB C
Mouse anti-RyR1	1:500	DSHB 34C
Rabbit anti-MEF2C	1:500	Cell Signaling 5030S
Rabbit anti-STIM1 (C-terminal)	1:500	Sigma S6197
Rabbit anti-MyoD1	1:200	Cell Signaling D8G3
**Secondary antibodies:**		
Alexa Fluor® 488-conjugated goat anti-mouse IgG (H + L)	1:1000	Life Technologies A11029
Alexa Fluor® 546-conjugated goat anti-mouse IgG (H + L)	1:1000	Life Technologies A11030
Alexa Fluor® 546-conjugated goat anti-rabbit IgG (H + L)	1:1000	Life Technologies A11030

### Nuclei segmentation analysis

Nuclei were detected based on their DAPI fluorescent channel using Cellpose 0.1.0.1 pretrained model [14], with automated detection of the diameter. Then the segmentation masks and the acquired images were processed with Matlab 2020b. Briefly, myocyte enhancer factor 2C (MEF2C) or Myogenic differentiation 1 (MyoD)-positive nuclei were analyzed if their individual area was between 40 and 200 μm^2^. Nuclei were considered as part of a cluster if their nearest neighbor distance was below 3 μm border-to-border. For each isolated nucleus (*i.e.,* not part of a cluster), orientation in the field of view and eccentricity (that informed about the shape of the nuclei) were computed. Nuclei detection and segmentation was performed by Nicolas Liaudet from the Bioimaging core facility of the University of Geneva (https://www.unige.ch/medecine/bioimaging).

### Calcium measurements

Myotubes were loaded with Cal520-AM (5 μM, AAT Bioquest®) and 0.1% Pluronic F-127 (Invitrogen, cat. No P3000MP), in the dark at 37 °C in Ca^2+^-containing solution. Following an incubation of 90 min, cells were washed and kept for 30 min to allow de-esterification of the dye. Fluorescence was recorded using a Zeiss Axio Observer A1 microscope equipped with a Lambda XL illumination system (Sutter Instrument, Novato, CA, USA). The excitation wavelength was 480 nm (ET480/20×; Chroma), and emission was collected through a T505lpxr dichroic mirror (Chroma) and a 510WB40 filter (Omega Optical) by a cooled 16-bit CMOS camera (pco.Edge sCMOS, Visitron Systems, Puchheim, Germany). An attenuation filter was added at the excitation side to limit phototoxicity (694/SP BrightLine HC shortpass filter, AHF analysentechnik AG, Germany). Acetylcholine (10 μM, A2661, Sigma-Aldrich) or electrical stimulations (see below) were applied to elicit Ca^2+^ responses. Image acquisition was performed at 3.3 Hz with the VisiWiew software, version 4.4.0.11 (Visitron Systems, Puchheim, Germany). The fluorescence intensity was expressed as F/Fo to normalize the data.

### Electric field stimulations

A chamber containing platinum electrodes (RC-37FS, Cat.64-0366, Warner Instruments) was inserted into the culture dish. For the experiments performed with cells grown on the nanopattern surface, the electrodes were oriented in a transversal direction compared with the long axis of the cells. The chamber was connected to an electric field stimulator (A310 Accupulser, World Precision Instruments), where the parameters of the stimulation were set up, and to an electric field isolator (A385 Stimulus isolator, World Precision Instruments) to supply a constant current.

Myotubes were stimulated with repetitive bursts of 1 s at 10 Hz every 10 s (each stimulation lasts 2 ms), at 70 V for 3 min. Three to four rounds of stimulations were performed on each cell culture. Stimulations were performed on a medium containing (mM) 135 NaCl, 5 KCl, 1 MgCl_2_, 10 Hepes, 10 glucose, 0.050 EGTA, and 2 CaCl_2_, pH 7.4 (NaOH). The analysis of Ca^2+^ transient parameters was performed with MatLab (R2020b).

### Quantitative and conventional real-time quantitative polymerase chain reaction (RT-PCR)

RNA was isolated using TRI Reagent Solution (AM9738, Invitrogen™) from myoblasts, myotubes differentiated for 4 and 10 days, and from adult muscles, according to the manufacturer’s instructions. Quantification of the samples, quality control, reverse transcription, and real-time quantitative polymerase chain reaction (RT-qPCR) were all performed at the iGE3 Genomic Platform of the University of Geneva (https://ige3.genomics.unige.ch/). Following the measurement of sample concentration, quality of the RNA was determined using the Agilent RNA 6000 Nano Kit and analyzed with the Agilent 2100 Bioanalyzer instrument (Agilent Technologies, Germany). From the total RNA, 0.5 μg was reverse transcribed with the PrimeScript™ RT reagent kit (TaKaRa, Bio Company, Japan) according to the manufacturer’s instruction. The expression of genes listed in Table 3 was evaluated during the differentiation and maturation of the myotubes and compared with the expression in the adult tissue. RT-qPCR was performed on 7900HT instrument (Applied Biosystems™). Raw threshold-cycle (Ct) values obtained with SDS 2.2 (Applied Biosystems™) were imported into Excel. Normalization factor and fold changes were calculated using the GeNorm method [15]. The level of gene expression was normalized for two housekeeping genes (*B2M* coding for β2 microglobulin and *EEF1A1*). Fold changes obtained for each condition were normalized to the adult tissues.

**Table 3:** List of primers

	Proteins	Names	Sequences
**Genes**			
CACNA1S	Cav1.1α1s	26-35F	CAC CTC CTC CTA CTT TGA ATA
		26-35R	AGA ACT TCC CAA AGC CCA GA
		E27F	GCT CAT GGC CTT CAA GG
		ex31R	TGA CGA TGA GCA GAG CC
RYR1	RyR1	ex-102-103-27F	TGG CCA TCA TCC AGG GTC T
		ex-102-103-77R	GGT CTC GGA GCT CAC CAA AAG
ATP2A1	SERCA1	ex-15-16-29F	CAG TGG CTG GCT CTT CTT CC
		ex-15-16-79R	GCA CCC ACA TAG CCC CC
ATP2A2	SERCA2	ex-3V-871F	CCT TGA GGA CTC TGC CAA CTT T
		ex-3V-921R	ACG AAG GTC AGA TTG GTC TCA TATT
MYH1	MyHC-2X (fast)	ex-39-40-48F	CAA GCT GAA GAA GCG GAG GA
		ex-39-40-98R	GCG GAA TTT GGA GAG GTT GAC
MYH2	MyHC-2A (fast)	ex-37-38-64F	AAA CTG GAG GCC AGG GTA CG
		ex-37-38-114R	TTG CTC ACT CTC AAC CTC TCC TT
MYH3	MyHC-3 (embryonic)	7F	TCA GAA GCC GAT TCT ACA TGG AC
		57R	ACA ACT TAG CGG CAC TTG GG
MYH7	MyHC-7 (slow)	ex-37-38-66F	AGG AGC TCA CCT ACC AGA CGG
		ex-37-38-116R	GCA GCC GCA GCA GGT TT
**Housekeeping genes**			
β2-microglobulin			TGC TCG CGC TAC TCT CTC TTT
			TCT GCT GGA TGA CGT GAG TAA AC
EEF1A1			AGC AAA AAT GAC CCA CCA ATG
			GGC CTG GAT GGT TCA GGT A

For conventional PCR, cDNA from myotubes differentiated for 4 and 10 days and from adult muscle were used. DNA fragments of Cav1.1 splice variant, Cav1.1a (adult; 350 bp) and Cav1.1e (embryonic; 250 bp), were separated in 2.2% agarose gel electrophoresis. Each DNA fragment was further sequenced by Fasteris DNA Sequencing Service (Fasteris SA, Switzerland).

### Statistics

For the analysis of nuclei clusters and shape, statistical analysis based on Wilcoxon rank sum tests were conducted on the median number of nuclei in clusters and on the median eccentricity of each acquisition. The null hypothesis between nuclei cultured on nanopattern and flat surfaces was considered as rejected if the *p* values were below the significant level of 0.05. For all other experiments, data are mean ± SEM, and the statistically significant differences were determined using a Student *t* test, where ^*^ *p* < 0.05, ^**^ *p* < 0.01, ^***^ *p* < 0.001, and ^****^ *p* < 0.00001.

## Results

### Myotube differentiation on flat versus striated surfaces

**Fig. 1:**
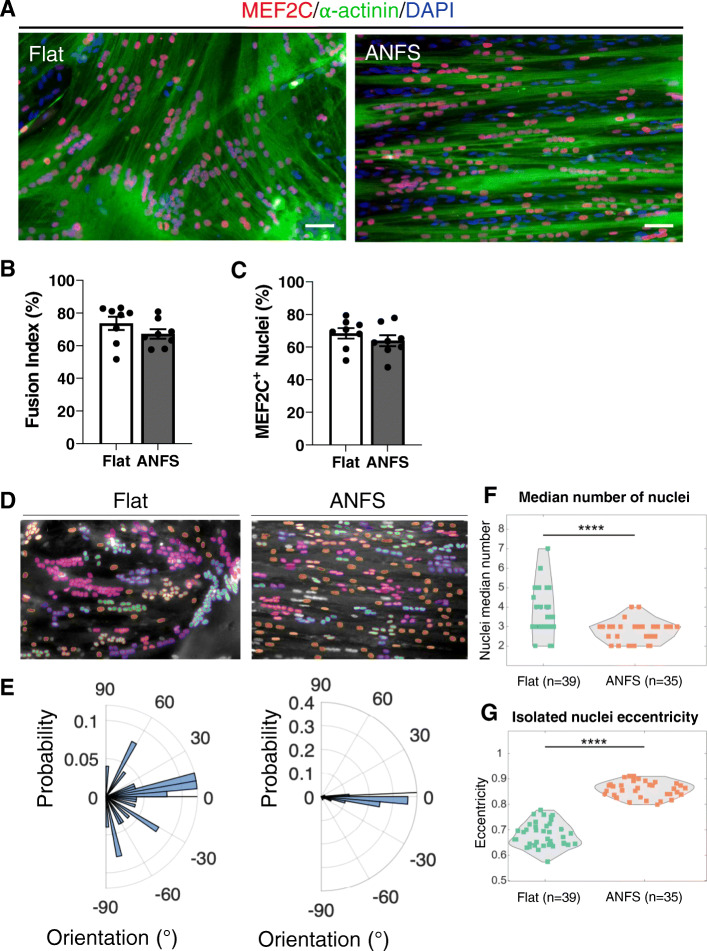
Nanopattern surface promotes myotube and nuclei alignment. **a** Immunofluorescence images of 4-day-old myotubes on flat (left panel) or nanopatterned (ANFS, right panel) surface. Myotubes were stained using antibodies against α-actinin (green) and MEF2C (red) together with a DAPI staining (blue). Scale bar: 50 μm. **b,c** Quantification of fusion index (**b**) and MEF2C-positive nuclei (**c**). Data were obtained from four independent experiments, and each dot is the mean of 3 fields/dish. Error bars are Mean ± SEM. **d** Nuclei detection based on a pretrained model on flat (left panel) and on nanopattern (right panel) surface. Each cluster of nuclei is depicted with the same color code. **e** Corresponding distribution of the nuclei orientation on flat (left panel) and on nanopattern surface (right). **f–g** Median number of nuclei in cluster (**f**) and median eccentricity of the isolated nuclei (**g**). Perfect round nuclei would have a value of 0, and elongated nuclei have an eccentricity value closer to 1. Each dot is the median value of a field, from four to five independent experiments. Statistical analysis is based on Wilcoxon rank sum test: ^****^ *p* = 5.76 × 10^-5^ < *α* = 0.05, *z* = 4.023; ^****^ *p* = 1.54 × 10^-13^ < *α* = 0.05, *z* = − 7.384

We first evaluated the benefit of growing and differentiating human primary myoblasts on a nanopattern surface (ANSF, see Methods section) compared with those on a flat surface. We analyzed the level of myotube differentiation after 4 days in DM, by immunolabeling the cells with α-actinin and the transcription factor MEF2C (Fig. 1a). The fusion index (number of nuclei within myotubes/total number of nuclei) and the percentage of MEF2C positive nuclei were similar between the two surfaces (Fig. 1b, c). However, organization of myotubes was notably different. On the flat surface, myotubes were mainly randomly oriented, while on the nanopattern surface, they were tightly aligned parallel to the striations (Fig. 1a). The position and the shape of the nuclei were also different (Fig. 1d, e): nuclei were frequently found as aggregates of up to 20 nuclei on flat surface (median value between 2 and 7), while the aggregates contained less nuclei (up to 8) on the nanopattern surface (median from 2 to 4; Fig. 1f), and the nuclei displayed predominantly a linear arrangement along the axis of the cells (Fig. 1e). As well, nuclei of cells grown on the nanopattern were more elongated (eccentricity close to 1), as compared with nuclei of cells grown on a flat surface (Fig. 1g). As dispersion and alignment of myonuclei are reminiscent of the sequence of events taking place during myotube maturation [16], these results suggested that the maturation of myotubes is more advanced when grown on a striated surface than on a flat surface.

To further characterize the differentiation process on both surfaces, we used immunostaining against the transcription factors paired box 7 (Pax7) and MyoD to evaluate the proportion of reserve cells in our two cultures (Supplemental Fig. 1A). During the early step of differentiation *in vitro*, reserve cells escape the terminal differentiation and commit to a quiescent state [17]. These cells express Pax7, but not MyoD (Pax7^+^/MyoD^-^), while myotubes express MyoD and downregulate Pax7 (Pax7^-^/MyoD^+^). We did not observe a difference between surfaces in the percentage of Pax7-positive cells (Supplemental Fig. 1B), indicating that the striated surface did not interfere with the establishment of reserve cells. MyoD-positive nuclei were, however, slightly more frequent on the striated surface (Supplemental Fig. 1C). Hence, the nanopatterned striated surface seems to favor specifically myotube maturation but not the entire process of myogenesis.

### Ca^2+^ response to electrical field stimulation on flat versus striated surfaces

**Fig. 2:**
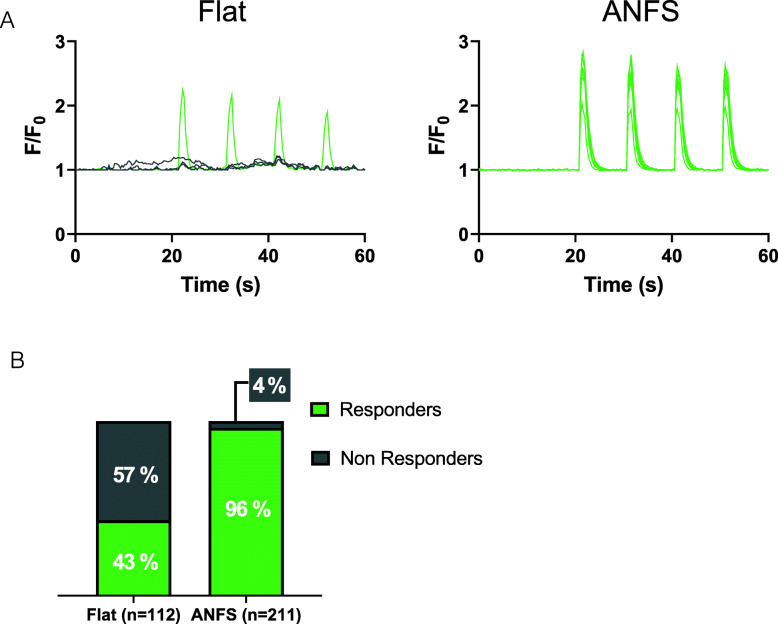
The nanopattern surface improves Ca^2+^ responses to electric field stimulation. **a** Four-day-old myotubes were loaded with 5 μM Cal520-AM to detect changes in cytosolic Ca^2+^ concentration upon 1 min of electric field stimulation (bursts of 1 s at 10 Hz, every 10 s). Representative recordings of myotube responses on flat and striated surfaces (ANFS). The fluorescence intensity was expressed as F/F_0_. **b** Cell responses to stimulations were classified based on the amplitude of the first peak: non-responders, amplitude < 0.5; responders, amplitude > 0.5. Bar charts represent the percentage of each category of responses on the two surfaces, flat and nanopattern (ANFS). Data were obtained from four independent experiments

We then investigated whether aligned myotubes obtained from the striated surface were functionally more advanced compared with those grown on a flat surface. To this end, we recorded Ca^2+^ responses after electrical field stimulation to assess the level of EC coupling. Four-day-old myotubes were electrically stimulated by repetitive bursts (1 s at 10 Hz, every 10 s) for 3 min. Myotubes grown on either surface responded by transient cytosolic Ca^2+^ elevations after stimulation. To note, while less than 45% myotubes cultured on a flat substrate displayed Ca^2+^ elevation upon stimulation, this percentage increased to more than 95% when the myotubes were grown on nanopattern (Fig. 2a, b). We cannot exclude that a small percentage of myotubes grown on a flat surface did not display a Ca^2+^ response to electrical field stimulation because of a suboptimal orientation of the cell compared with the electrodes. However, as the myotubes on the flat surface grow in all directions, they are less sensitive to an optimal orientation, and this could not explain the low percentage of responding cells that we observed on the flat surface. Hence, this increased response to electrical field stimulations strongly suggests that myotubes grown on a striated pattern are not only better organized but are also functionally more mature.

### Gene expression during maturation on striated surface

The poor myotube organization and their progressive detachment on a flat substrate prevent keeping our cultures for more than 4 to 5 days. On the contrary, myotubes adhere better when cultured on nanopattern, and after addition of a second thick layer of Matrigel, we could keep them for up to 10–12 days in culture and study accurately their maturation. Under these conditions, the width of the myotubes remained stable over time (19.7 ± 1.1 μm to 19.8 ± 0.7 μm at 4 and 10 days, respectively; data not shown).

**Fig. 3:**
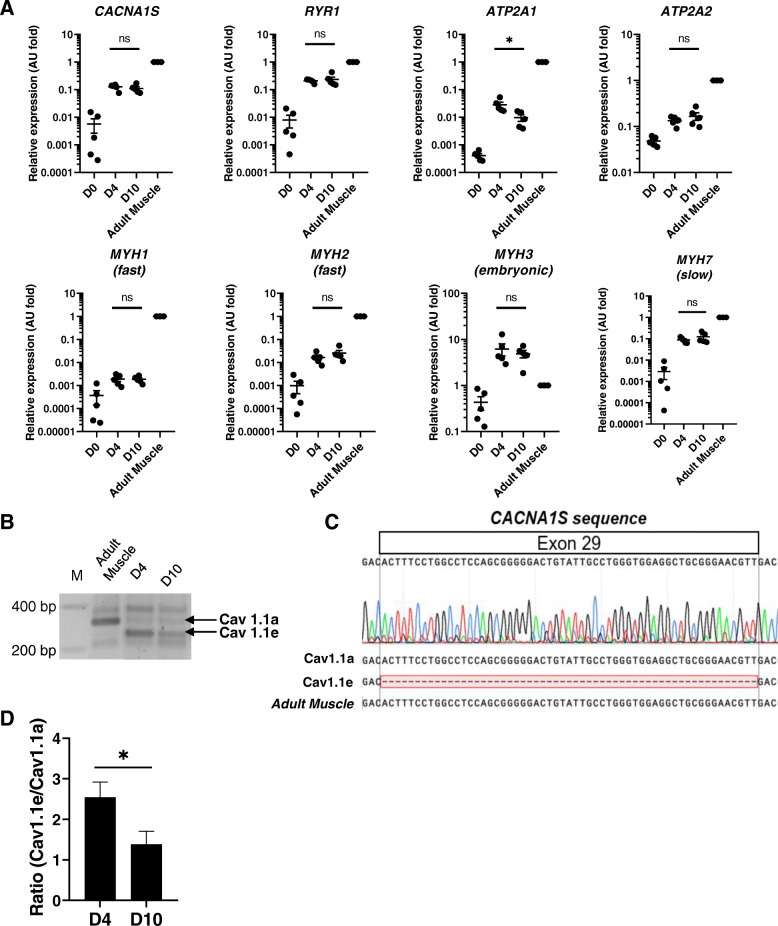
Expression of EC coupling-associated proteins and myosin heavy chain during myotube maturation. **a** Transcript levels of genes encoding for proteins involved in muscle contraction and EC coupling were quantified by RT-qPCR in adult muscle (three donors) and in myoblasts (D0), 4-day-old (D4), and 10-day-old (D10) myotubes (cultures from five donors). *CACNA1S* (Cav1.1), *RYR1* (RyR1), *ATP2A1* (SERCA1), *ATP2A2* (SERCA2), *MYH1* (MyHC-2X), *MYH2* (MyHC-2A), *MYH3* (MyHC-3 embryonic) and *MYH7* (MyHC-7 slow). Transcript levels are relative to adult muscle transcript content. Results are mean ± SEM. *A.U.*, arbitrary units. Each dot represents one independent experiment. **b** PCR products of the two variants of Cav1.1, embryonic (Cav1.1e) and adult (Cav1.1a) detected in 4- and 10-day-old myotubes and in human adult muscle. *M*, marker of molecular weights. **c** PCR product sequencing of *CACNA1S* exon 29, specific of the adult isoform of Cav1.1 (Cav1.1a) but absent in the embryonic isoform (Cav1.1e). **d** Quantification of the ratio between Cav1.1e and Cav1.1a expressions in 4- and 10-day-old myotubes. Error bars represent mean ± SEM

To obtain a broad overview of the myotube maturation achieved on nanopattern surface, we analyzed several parameters, the first one being the gene expression of Ca^2+^-handling proteins (Cav1.1α1s, RyR1, and sarco-endoplasmic Ca^2+^ ATPase (SERCA)1/2) and myosin heavy-chain isoforms, by RT-qPCR in cultured myotubes (at 4 and 10 days) and, for comparison, in adult muscle sample. As expected, the expression of all these genes increased between myoblasts and differentiated myotubes, while their expression remained stable between 4 and 10 days in culture (Fig. 3a). Transcript levels of *CACNA1S*, *RYR1*, *MYH7* (encoding the isoform expressed in slow fibers), and *ATP2A2* (encoding SERCA2) were less than 10-fold lower in myotubes compared with those in adult fibers. On the contrary, genes encoding proteins abundant in fast fibers (*MYH1*, *MYH2,* and *ATP2A1*) were between 10- and more than 100-fold less expressed in myotubes than in adult tissue. Transcript levels of *MYH3* (coding the embryonic myosin heavy chain) slightly decreased between 4- and 10-day old myotubes, and they were higher in myotubes than in the adult tissue (Fig. 3a). In parallel, we assessed the expression of the embryonic and adult isoforms of the Cav1.1α1s subunit, namely the Cav1.1e and the Cav1.1a, respectively. Immature myotubes express predominantly the Cav1.1e isoform (that lacks exon 29), while it represents less than 10% of the transcripts in differentiated muscle [18]. Using conventional PCR analysis, we detected two bands corresponding to the embryonic and adult isoforms of Cav1.1 both at 4 and 10 days, while on the adult tissue, only the Cav1.1a was expressed (Fig. 3b). Sequencing the PCR product confirmed that the lower band found in myotubes lacks exon 29 (Fig. 3c). Furthermore, the ratio of Cav1.1e and Cav1.1a expression decreased between 4- and 10-day old myotubes (Fig. 3d), highlighting a shift towards the expression of the adult Cav1.1 channel with time in culture.

### Organization of muscle-specific proteins and Ca^2+^ responses

**Fig. 4:**
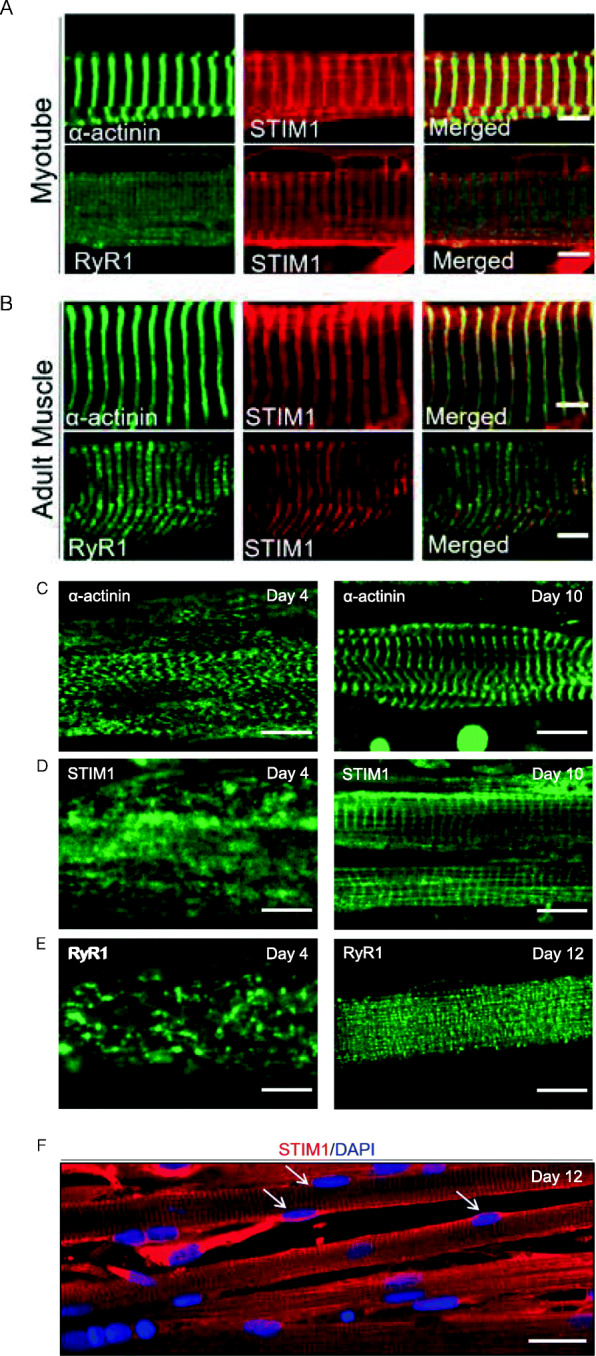
Organization of muscle-specific proteins and of nuclei. Representative images of 10-day-old myotubes cultured on nanopattern surface (**a**) and adult human muscle tissue (**b**) immunolabeled for α-actinin (green) and STIM1 (red) (upper lines) and for RyR1 (green) and STIM1 (red) (lower lines). Scale bar: 5 μm. **c–e** Organization of α-actinin, STIM1, and RyR1 during maturation. Representative images of 4-, 10-, and 12-day-old myotubes immunolabeled for α-actinin (**c**) STIM1 (**d**), and RyR1 (**e**). At 4 days, α-actinin is already perpendicularly oriented, followed at 10 days by STIM1, and in some cells by RyR1 at 12 days. Scale bar: 5 μm. **f** Representative image of 12-day-old myotubes immunolabeled with STIM1 together with DAPI. White arrows point to peripheral nuclei. Scale bar: 20 μm

Skeletal muscles have a highly organized internal architecture with striated pattern of most of the muscle-specific proteins. To evaluate how mature are myotubes grown on the nanopattern surface, we performed immunostaining of α-actinin, RyR1, and the SR Ca^2+^ sensor stromal interaction molecule 1 (STIM1) after 10–12 days of differentiation. As shown on Fig. 4a, the staining of all these proteins appeared striated, and the distance between two *z* lines was of around 2.7 μm, close to what is found in the adult human muscle (2.5 μm; Fig. 4b and Supplemental figure 2). Over time, the typical perpendicular alignment of α-actinin appeared first (at around 4 days) followed by STIM1 (at 8–10 days) and eventually RyR1 (Fig. 4c–e). The striations of α-actinin and STIM1 were observed in a consistent way, while the RyR1 doublets were seldom obtained (in about 20% of the cultures) after 10 days of myotube maturation. We further noticed that on myotubes at that stage, the nuclei were mostly localized at the cell periphery (Fig. 4f). Importantly, already after 6 days in DM, the myotubes displayed frequently spontaneous contractions, in around 30–40% of the cultures (Movie 1).

**Fig. 5:**
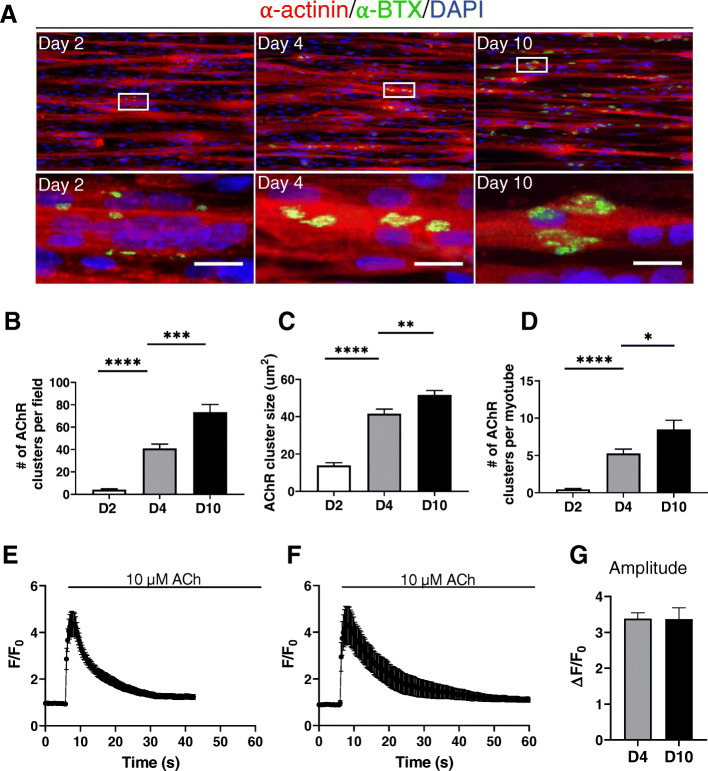
Organization of AChR clusters during maturation and Ca^2+^ response to acetylcholine. **a** Representative images of 2-, 4-, and 10-day-old myotubes cultured on nanopattern surface and immunolabeled for α-actinin (red) and α-bungarotoxin (α-BTX, green) together with a DAPI staining (blue). Lower panels are enlargement of the white rectangles. Scale bar: 20 μm. **b, c, d** Quantification of the average number of acetylcholine receptors (AChR) clusters per field (**b**)*,* AChR cluster size (**c**), and of the average numbers of AChR clusters per myotube (**d**). Data were obtained from three independent experiments. Error bars are mean ± SEM. **e−f** Myotubes were loaded with 5 μM Cal520-AM to measure changes in cytosolic Ca^2+^, and stimulated with 10 μM of acetylcholine (ACh). Traces represent the mean ± SEM of Ca^2+^ responses of myotubes differentiated for 4 (**e**) and 10 days (**e**). **g** Statistical evaluation of the amplitude of the peak following ACh stimulation. Error bars are mean ± SEM

Next, we analyzed the presence of AChR clusters at different times of maturation. Both number and size of clusters significantly increased within 2, 4, and 10 days of culture (Fig. 5a–d). The shape of the clusters is known to change during maturation, with the emergence of branched structures with time [19]. We detected only very few clusters with such rearrangement of the AChR after 10 days of maturation, most of the clusters being oval-shaped. However, AChR were functional, as shown by the rapid cytosolic Ca^2+^ elevation elicited by application of 10 μM acetylcholine (Fig. 5e–g). By comparison, on the flat surface at day 4 of differentiation, the AChR were more dispersed on the myotubes and did not form proper clusters (Supplemental Figure 3). In addition, only 64% of the myotubes displayed a Ca^2+^ response when stimulated with 10 μM ACh, compare with the 97% of responding myotubes grown on the nanopattern surface (Supplemental Figure 3).

**Fig. 6:**
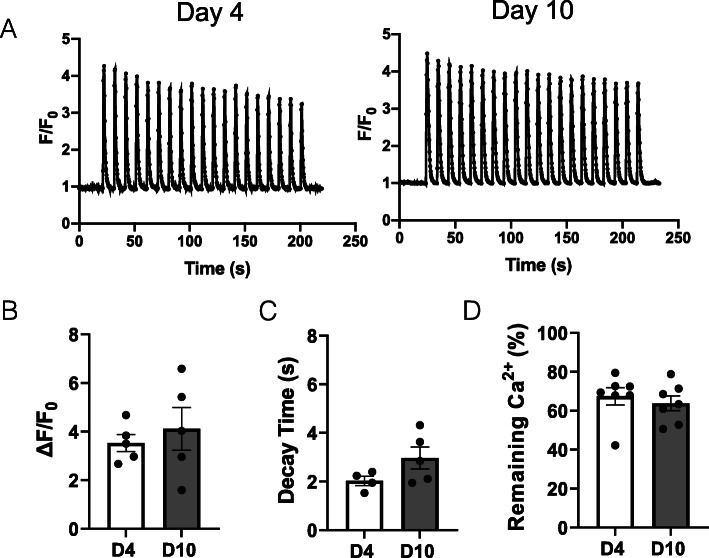
Ca^2+^ transients upon electric field stimulation. **a** Representative traces of cytosolic Ca^2+^ transients generated by repeated burst of 1 s at 10 Hz, every 10 s for 3 min in 4-day (left panel) and 10-day (right panel)-old myotubes, differentiated on nanopattern surface. Statistical evaluation of the amplitude (**b**) and the decay time (**c**) of the first Ca^2+^ transient, and the percentage of remaining calcium at the end of 3-min stimulation (**d**). Data were obtained from four independent experiments, and each dot is a mean of 4−6 myotubes on a field. Error bars are mean ± SEM

Lastly, we assessed Ca^2+^ responses to electrical stimulations. Four- and 10-day-old myotubes were stimulated for 3 min with repetitive bursts every 10 s (Fig. 6a). The amplitude and the decay time of the first cytosolic Ca^2+^ elevation were very similar at early and later stages of maturation (Fig. 6b, c). As well, the amplitude of the last transient compared with the first one (percentage of remaining Ca^2+^), that depicts the sustained nature of the response was also not significantly different between the two time points (Fig. 6d). Hence, myotubes already displayed robust EC coupling after 4 days when cultured on nanopattern striated surface.

## Discussion

We reported here a simple *in vitro* culture model of human primary myoblasts that presents greater level of myotube maturation compared with commonly used 2D cultures.

Our culture model combined various procedures that foster myotube maturation. First, cells were grown between two layers of Matrigel that provided an extracellular matrix limiting cell detachment, as previously reported [13]. Second, cells were grown on a striated pattern surface that favors alignment and maturation of myotubes [11, 20, 21]. This surface is a fabricated pattern with grooves and ridges of 800-nm width and 600-nm depth, dimensions that were previously described to favor myotube maturation [20], even if larger striations (up to 1600 nm) also led to an enhanced maturation [21]. In addition, we added during the initial 4 days of differentiation an inhibitor of TGFβ receptor, a procedure that was shown to improve cell fusion [22], *MYH* expression, and sarcomere organization [23, 24].

As expected, the myotubes grown on a striated surface were very well aligned, with nuclei being also more elongated, initially aligned in the middle of the myotubes while moving at the cell periphery with time in culture, hence recapitulating the progression of nuclei movement occurring *in vivo* [16]. Yet, we did not notice differences, neither in fusion index nor in percentage of MEF2C-positive nuclei, between flat and grooved surfaces after 4 days of differentiation. This is somehow in contradiction with what was previously reported, where differentiation performed on striated surfaces enhanced the fusion index [20, 21]. It should be pointed out, however, that human primary cells cultured in our laboratory has a high fusion index (around 60%), higher than what was reported in the abovementioned studies. In line with the fusion index, the percentage of Pax7-positive cells was also similar between the two surfaces. Hence, our culture system did not favor the initial steps of differentiation but presents the advantage to promote cell alignment and to maintain cells for longer time in culture allowing their maturation.

We analyzed then several parameters during 10–12 days in culture: the expression level of muscle-specific genes, the internal organization of the myotubes, and their Ca^2+^ responses to ACh and electrical stimulations, in order to provide a broad overview of the processes taking place over time, both morphologically and functionally.

Although the myotube diameter remained stable during time in culture, the number and size of AChR clusters increased between 2 and 10 days of maturation. At 10 days of differentiation, almost all myotubes displayed clusters of AChR, which reflects a greater maturation of myotubes compared with conventional 2D cultures where only a minority of myotubes have AChR clusters after 2 weeks in culture [8]. The clusters remained however essentially homogenous, without a sign of lacunarity or pretzel structures, like it was reported in 3D culture [8] or on surfaces coated with various laminin [25], morphological changes that reflect the maturation of AChR clusters [19, 26]. AChRs were however functional as shown by the Ca^2+^ elevations elicited by ACh stimulation. The amplitude of the response was akin to the one elicited by electrical stimulation, in line with what was reported on human 3D myobundles [27]. On the contrary, 4-day-old myotubes grown on a flat surface have a diffuse pattern of AChR and not all myotubes responded to ACh stimulation by a Ca^2+^ elevation.

Interestingly, the genes expressed predominantly in slow fibers (*MYH7* and *ATP2A2*) were expressed in 10-day-old myotubes at levels closer to adult tissue, than the ones expressed in fast fibers (*MYH1*&*2* and *ATP2A1*). Hence, even if there was a mixed expression of genes from slow and fast fibers, like it is frequently observed in cultured myotubes [28, 29] [7], myotubes appear to mature preferentially towards a slow phenotype. This slow phenotype resembles the one observed during the establishment of a human muscle cell line HMCL-7304 [30], with a higher abundance of slow *MYH*. This slow phenotype is however different to what is observed *in vivo* when regeneration happens in the absence of innervation, where fibers follow what is called the “default pathway” toward a fast phenotype [31]. The reasons of this maturation towards a slow phenotype are unclear, but this may, at least partially, explain the kinetics of the Ca^2+^ responses triggered by ACh or electrical stimulation that we measured. Indeed, even if myotubes in culture are known to have slower Ca^2+^ recovery phase than adult fibers [32], we were anticipating a faster decay time associated with enhanced maturation, which was not the case. Certainly, the slow Ca^2+^ kinetics of the response also reflects the fact that the system did not sufficiently mature to have an obvious impact on the Ca^2+^ repumping efficiency. During muscle development, a splice variant of Cav1.1 lacking exon 29, namely Cav1.1Δ29 or Cav1.1e is expressed, characterized by distinct biophysical properties compared with the adult Cav1.1 isoform: a higher Ca^2+^ current amplitude together with a 30-mV left shift of the activation potential [18, 33]. The expression of Cav1.1e was also reported in human myotubes in culture, leading us to evaluate its expression. Our results show that the embryonic Cav1.1e is expressed both at 4 and 10 days, and interestingly, the ratio between the two, embryonic and adult isoforms, decreased with time in culture, confirming an enhanced fiber maturation at 10 days.

We also assessed the organization level of several proteins over time in culture. As already reported [34], the transverse alignment of α-actinin occurred rapidly after the induction of differentiation. Indeed, after 4 days in culture, a substantial number of cells displayed this typical arrangement, and the striations get sharper with longer time in culture. The distance between two Z lines in 10-day-old myotubes was around 2.7 μm, close to the size of adult human sarcomeres (2.5 μm). Furthermore, after 7–8 days in culture, the SR resident Ca^2+^ sensor STIM1 also appeared striated, with an important level of colocalization on the Z lines. This localization of STIM1 is consistent with findings obtained in muscle adult tissue, where STIM1 was reported to be present both at a region corresponding to the I band (around the Z lines), as well as on the longitudinal SR [35] [36, 37]. A proper RyR1 localization in doublet was however infrequently obtained, even after 12 days of maturation. On the few cases where such matured arrangement of RyR1 was observed, it always took place after STIM1 was perpendicularly organized. Even if RyR1 doublets were seldom observed, the high level of internal organization achieved within 10 days was to our knowledge not obtained with human cells cultured in 2D. Advanced maturation were obtained with human iPSC but after a significantly longer time (50 days to obtain titin striations [6], or after 2 weeks to obtain α-actinin striations of myotubes derived from induced myogenic progenitor cells [7]. Hence, the advantage of growing the myotubes on a striated surface, beside the improvement of cell adhesion, is that myotubes remain thin and elongated which likely promotes their internal organization and thus maturation.

Finally, we assessed the ability of myofibers to elicit cytosolic Ca^2+^ transients upon electrical field stimulation. Already at 4 days of culture, electrical stimulations resulted in large Ca^2+^ transients of almost all myotubes, highlighting a functional EC coupling. This time frame is rapid if we compared it to the 8 days needed to obtain 50% of myotubes responding to electrical stimulation with classical 2D culture of human cells [32]. We analyzed at 4 and 10 days of culture three parameters out of these Ca^2+^ recordings, the amplitude of the first transient, the kinetic of the Ca^2+^ recovery phase, and the sustained nature of the signals during the 3-min stimulation. None of these parameters significantly changed between 4 and 10 days, showing that even if the cells displayed a better internal organization at 10 days, this did not correlate with an improved Ca^2+^ signals. This is however in agreement with the expression levels of *RYR1*, *CACNA1S,* and *ATP2A1/2* that remain stable during the maturation period we analyzed.

## Conclusions

Overall, we proposed a 2D culture system of human myotubes that reach after 10 days a high level of maturation, with the presence of numerous functional AChR clusters, the expression of adult *MYH*, increased adult *CACNA1S* isoform expression, and a high level of internal fiber organization. Further improvements are however required to obtain myofibers that resemble more the adult organization and function. Nevertheless, this rapid and easy-to-perform 2D culture, which does not require sophisticated technology, presents certainly great advantages for further single-cell studies designed to investigate the processes of skeletal muscle maturation.

## Supplementary Information

**Additional file 1: Movie 1.** Spontaneous myotube contraction on 6-day old culture on nanopattern surface.

**Additional file 2: Supplemental Figure 1.** The reserve cell population is not affected by the surface pattern. **(A)** Immunofluorescence of nuclei of 4-day old myotubes on Flat (upper panel) or nanopattern surface (ANFS, lower panel). Cell nuclei were stained using an antibody against Pax7 (in green), MyoD (in red) and all nuclei were counterstained with DAPI (in blue). Scale bar: 50 μm. Images shown are representative of six independent experiments. **(B, C)** Percentage of Pax7 positive nuclei **(B)** and MyoD positive nuclei **(C)**. Error bars are Mean ± SEM. **Supplementary Figure 2.** Length of the sarcomere on culture myotubes and on adult tissue. **(A-B)** Representative images of 10-day old myotubes cultured on nanopattern surface **(A)** and adult human muscle tissue **(B)** Scale bar: 10 μm. **(C-D)** Corresponding line profiles (white dotted line) of α-actinin normalized fluorescence intensity. **Supplemental Figure 3.** Organization of AChR clusters and Ca^2+^ response to acetylcholine on flat and striated surface. **(A)** Representative images of 4-day old myotubes cultured on flat (left panel) and nanopattern surface (right panel) and immunolabeled for α-actinin (red) and α-bungarotoxin (α-BTX, green) together with a DAPI staining (blue). Scale bar: 50 μm. **(B)** Myotubes were loaded with 5 μM Cal520-AM to measure changes in cytosolic Ca^2+^ and stimulated with 10 μM of acetylcholine (ACh). Traces represent the mean ± SEM of Ca^2+^ responses of myotubes differentiated for 4 day on flat surface (left panel) and striated surface (right panel). **(C**) Statistical evaluation of the amplitude of the peak following ACh stimulation. Error bars are Mean ± SEM. **(D)** Bar chart representing the percentage of responding cells on both surfaces. Data were obtained from three independent experiments.

## Data Availability

The datasets used during the current study are available from the corresponding author upon request.

## Abbreviations

GM: Growth medium
DM: Differentiation medium
SR: Sarcoplasmic reticulum
EC: Excitation–contraction
MEF2C: Myocyte enhancer factor 2C
MyoD: Myogenic differentiation 1
Pax7: Paired box 7
RyR1: Ryanodine receptor 1
DHPR: Dihydropyridine receptor
STIM1: Stromal interaction molecule 1
ACh: Acetylcholine
AChR: Acetylcholine receptor
α-BTX: α-Bungarotoxin
SERCA: Sarco-endoplasmic Ca^2+^ ATPase
