# Supplementary material for: Nanopattern surface improves cultured human myotube maturation
Source: Skelet Muscle. 2021 May 5;11:12. doi: 10.1186/s13395-021-00268-3 (PMC8097894; doi:10.1186/s13395-021-00268-3)

Supplemental Figure 1

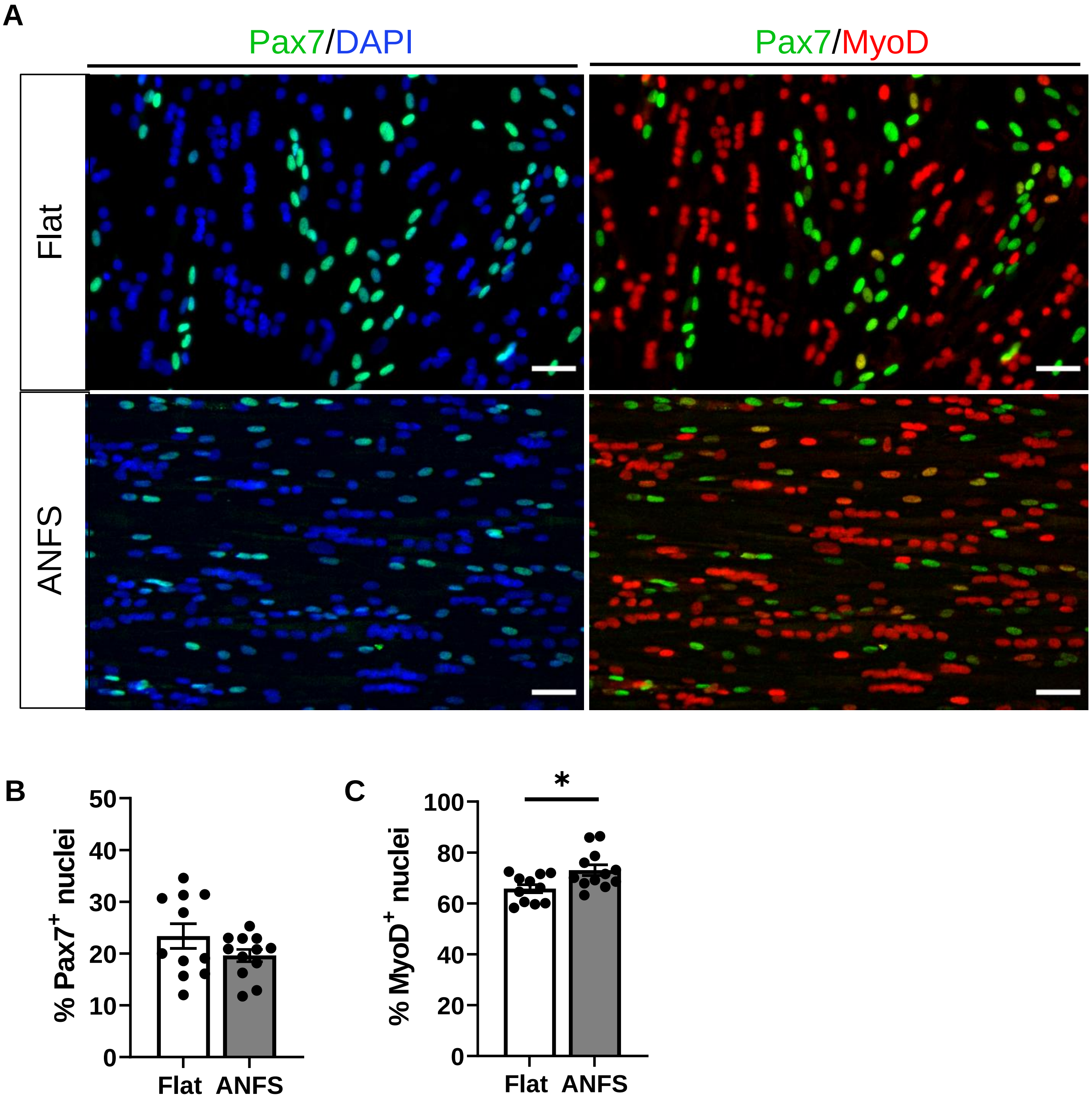

Supplemental Figure 2

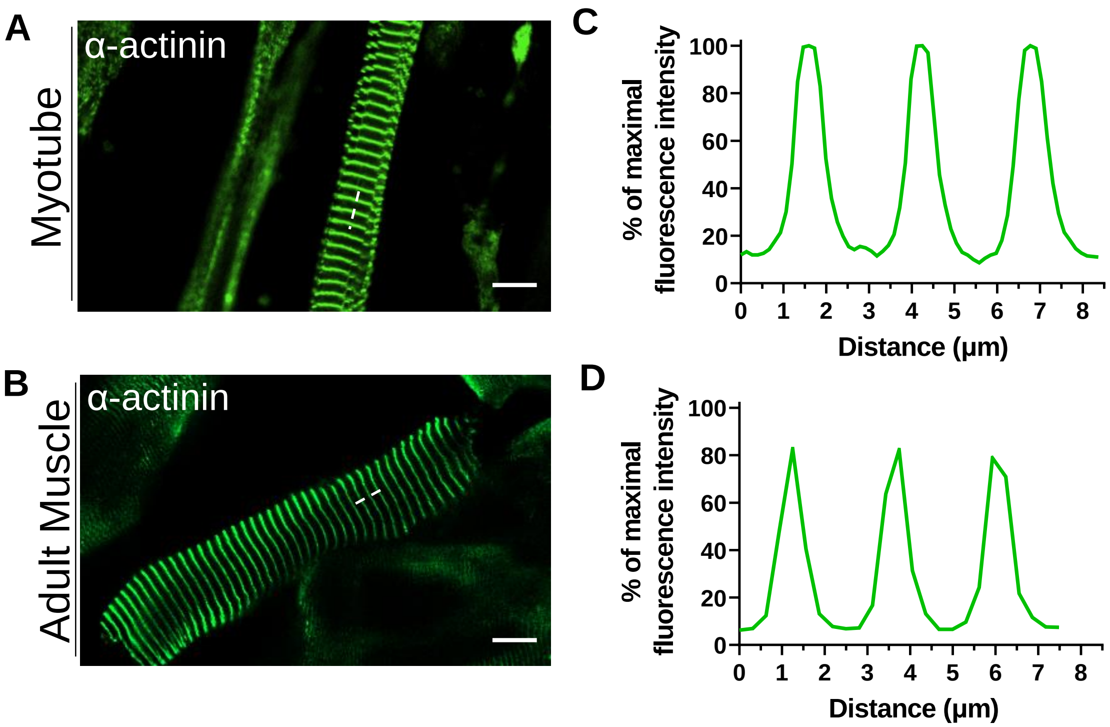

Supplemental Figure 3

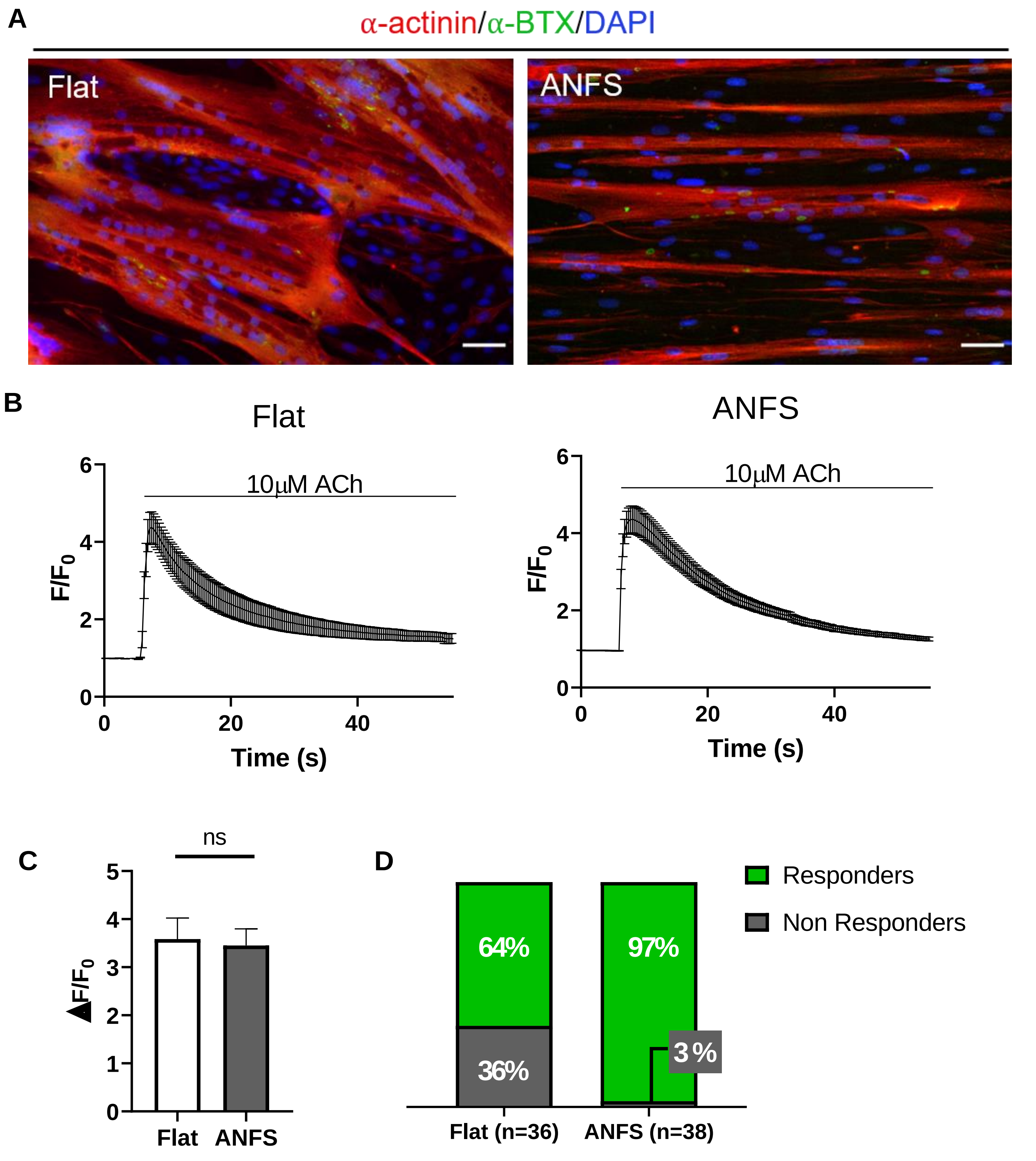

Supplement: Supplementary file 2 — Additional file 2: Supplemental Figure 1. The reserve cell population is not affected by the surface pattern. (A) Immunofluorescence of nuclei of 4-day old myotubes on Flat (upper panel) or nanopattern surface (ANFS, lower panel). Cell nuclei were stained using an antibody against Pax7 (in green), MyoD (in red) and all nuclei were counterstained with DAPI (in blue). Scale bar: 50 μm. Images shown are representative of six independent experiments. (B, C) Percentage of Pax7 positive nuclei (B) and MyoD positive nuclei (C). Error bars are Mean ± SEM. Supplementary Figure 2. Length of the sarcomere on culture myotubes and on adult tissue. (A-B) Representative images of 10-day old myotubes cultured on nanopattern surface (A) and adult human muscle tissue (B) Scale bar: 10 μm. (C-D) Corresponding line profiles (white dotted line) of α-actinin normalized fluorescence intensity. Supplemental Figure 3. Organization of AChR clusters and Ca2+ response to acetylcholine on flat and striated surface. (A) Representative images of 4-day old myotubes cultured on flat (left panel) and nanopattern surface (right panel) and immunolabeled for α-actinin (red) and α-bungarotoxin (α-BTX, green) together with a DAPI staining (blue). Scale bar: 50 μm. (B) Myotubes were loaded with 5 μM Cal520-AM to measure changes in cytosolic Ca2+ and stimulated with 10 μM of acetylcholine (ACh). Traces represent the mean ± SEM of Ca2+ responses of myotubes differentiated for 4 day on flat surface (left panel) and striated surface (right panel). (C) Statistical evaluation of the amplitude of the peak following ACh stimulation. Error bars are Mean ± SEM. (D) Bar chart representing the percentage of responding cells on both surfaces. Data were obtained from three independent experiments. [file 13395_2021_268_MOESM2_ESM.pdf]
